# Simultaneous Determination of the Traditional Herbal Formula Ukgansan and the In Vitro Antioxidant Activity of Ferulic Acid as an Active Compound

**DOI:** 10.3390/molecules23071659

**Published:** 2018-07-07

**Authors:** Yu Jin Kim, Soo-Jin Jeong, Chang-Seob Seo, Hye-Sun Lim, Eunjin Sohn, Jiyeon Yun, Bu-Yeo Kim

**Affiliations:** 1 Clinical Medicine Division, Korea Institute of Oriental Medicine, Daejeon 34054, Korea; jinjin0228@kiom.re.kr (Y.J.K.); qp1015@kiom.re.kr (H.-S.L.); ssen4022@kiom.re.kr (E.S.); jyeon7139@kiom.re.kr (J.Y.); buykim@kiom.re.kr (B.-Y.K.); 2 College of Pharmacy, Chungnam National University, Daejeon 34134, Korea; 3 Korean Medicine Life Science, University of Science & Technology, Daejeon 34113, Korea; 4 Herbal Medicine Research Division, Korea Institute of Oriental Medicine, Daejeon 34054, Korea

**Keywords:** Ukgansan, simultaneous determination, antioxidant, ferulic acid, neuroprotection

## Abstract

Ukgansan (UGS), a traditional herbal formula composing seven medicinal herbal plants, has been applied in Asian countries for treating neurosis, insomnia, and irritability. Here, the current study performed a simultaneous determination of the seven marker compounds (liquiritin apioside, liquiritin, ferulic acid, glycyrrhizin, decursin, decursinol angelate, and atractylenolide I) using high-performance liquid chromatography (HPLC), to establish quality control of UGS. A 70% ethanol extract of UGS and a mixture of the seven compounds were separated using a C-18 analytical column on a gradient solvent system of 1.0% (*v*/*v*) aqueous acetic acid and acetonitrile. Data were recorded at a UV wavelength of 250 nm for glycyrrhizin; 276 nm for liquiritin apioside, liquiritin, and atractylenolide I; and 325 nm for ferulic acid, decursin, and decursinol angelate. The results exhibited high linearity (correlation coefficient (*r*^2^) ≥ 0.9998) and proper precision (0.38–3.36%), accuracy (95.12–105.12%), and recovery (95.99–104.94%) for the seven marker compounds. The amount of the seven marker compounds at the concentrations from 0.190 to 16.431 mg/g. In addition, the current study evaluated the antioxidant effects of UGS by measuring their scavenging activities against the 2,2′-azinobis-(3-ethylbenzothiazoline-6-sulfonic acid) (ABTS) and 2,2′-diphenyl-1-picrylhydrazyl (DPPH) radicals using in vitro cell-free systems and observed its antioxidant activity. Among the seven components of the UGS extract, ferulic acid dramatically enhanced the scavenging of ABTS and DPPH radicals compared with other compounds. The concentrations of ferulic acid required for a 50% reduction (RC_50_) in ABTS and DPPH radicals were 16.22 μM and 41.21 μM, respectively. Furthermore, UGS extract exerted the neuroprotective effect and blocked the inflammatory response in neuronal hippocampal cells and microglia, respectively. Overall, the established method of HPLC will be valuable for improving the quality control of UGS extract, and ferulic acid may be useful as a potential antioxidant agent.

## 1. Introduction

Ukgansan (UGS), which is also called yokukansan in Japan and Yi-gan san in China, is a traditional Oriental herbal formula composing seven medicinal herbal plants including *Uncaria sinensis*, *Atractylodes japonica*, *Poria cocos*, *Bupleurum falcatum*, *Angelica gigas*, *Cnidium officinale*, and *Glycyrrhiza uralensis*. UGS has been utilized to manage various diseases such as neurosis, insomnia, and irritability in children and is a herbal medicine that is approved by the Ministry of Health, Labor and Welfare of Japan. Modern pharmacological and clinical studies have suggested that UGS has the potential to improve insomnia [1,2], borderline personality disorder [3], neuroleptic-induced tardive dyskinesia [4], drug-induced parkinsonism [5], cognitive impairment [6], and the behavioral and psychological symptoms of dementia (BPSD) [7,8,9]. It is well known that the occurrence of these diseases is closely related to oxidative stress [10,11,12]. Thus, antioxidant activity should be considered, in addition to the efficacy of therapeutic drugs.

The quality of herbal formulas depends on environmental factors, such as harvesting, storing, processing, and formulating methods. Because of their multiple components and the effect of the environment, quality assessment of the major components of herbal formulas is required for investigating their efficacy and safety [13]. Although the identification and simultaneous determination of the components of UGS using high-performance liquid chromatography (HPLC) and HPLC-Q-TOF-MS have been reported [14,15], there are few studies on simultaneous determination of the compounds in UGS using HPLC. Therefore, the current study conducted a simultaneous determination of the marker compounds of UGS for the quality control of UGS using a photodiode array HPLC detector (denoted the HPLC-PDA method), as it is the most popular analytical method. The HPLC-PDA method is a convenient and rapid analytical method to separate and identify the multiple compounds in herbal formulas [16,17].

In this study, simultaneous analysis of seven compounds (liquiritin apioside, liquiritin, ferulic acid, glycyrrhizin, decursin, decursinol angelate, and atractylenolide I) in a UGS extract was performed, and method validation was carried out using the HPLC-PDA method. Moreover, the antioxidant activities of the seven marker compounds were determined using in vitro radical scavenging assays and the biological effects in neuronal cell lines HT22 hippocampal cells and BV-2 microglia. A variety of drugs have shown the neuroprotective activity against damage-induced HT22 cells and the inhibitory effects on neuroinflammation in lipopolysaccharide-stimulated BV-2 cells [18,19,20].

## 2. Results

### 2.1. Optimization of HPLC Separation

**Figure 1:**
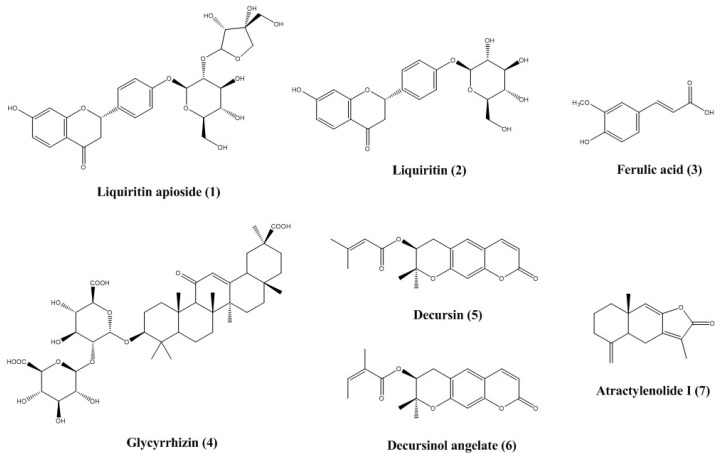
Chemical structures of the seven marker compounds of UGS.

**Figure 2:**
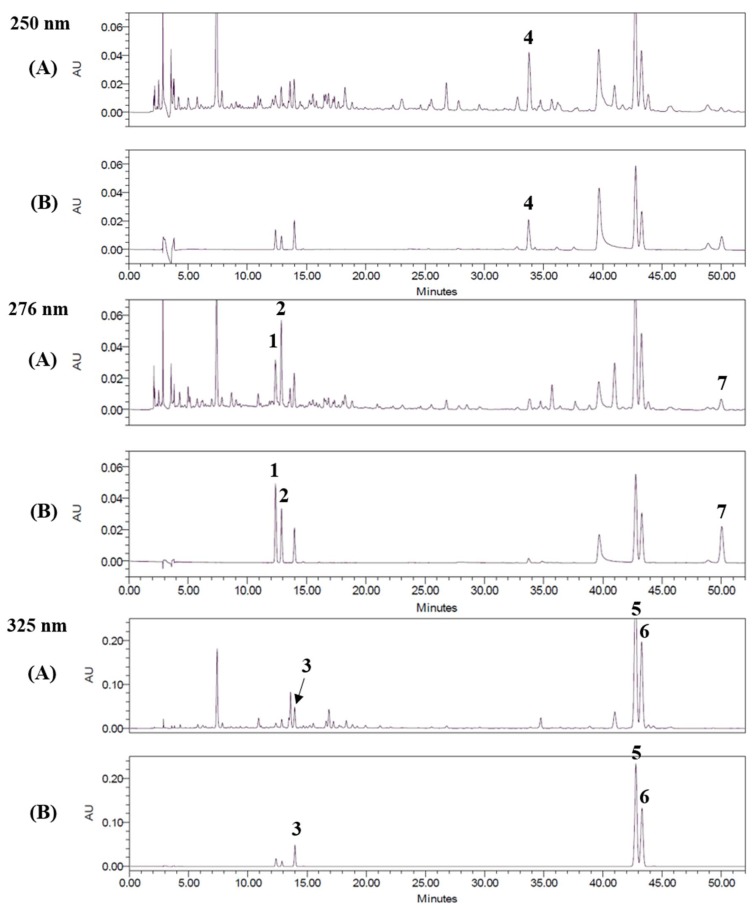
HPLC chromatograms of the 70% ethanol extract of UGS (**A**) and a standard mixture (**B**) at 250 nm, 276 nm, and 325 nm. Liquiritin apioside (**1**), liquiritin (**2**), ferulic acid (**3**), glycyrrhizin (**4**), decursin (**5**), decursinol angelate (**6**), and atractylenolide I (**7**).

HPLC analysis was performed for separating the seven marker compounds (Figure 1) from a 70% ethanol extract of UGS (Table 1). To establish an efficient separation of the seven compounds, various mobile phases were evaluated, including water, acetonitrile, and methanol with trifluoroacetic acid (TFA), acetic acid, and phosphoric acid. The results showed good separation chromatograms using mobile phases consisting of 1.0% (v/v) aqueous acetic acid (A) and acetonitrile (B). The UV wavelength used for quantitative analysis was 250 nm for glycyrrhizin; 276 nm for liquiritin apioside, liquiritin, and atractylenolide I; and 325 nm for ferulic acid, decursin, and decursinol angelate. Using the established methods of HPLC, the seven marker compounds were resolved within 50 min. The retention times of liquiritin apioside, liquiritin, ferulic acid, glycyrrhizin, decursin, decursinol angelate, and atractylenolide I were 12.36, 12.86, 13.95, 33.77, 42.74, 43.25, and 49.99 min, respectively. HPLC chromatograms of the UGS extract in 70% ethanol and the standard compound mixture are shown in Figure 2.

### 2.2. Regression Equation, Linearity, and Limits of Detection (LOD) and Quantification (LOQ)

The linear relationships between the concentrations (*x*, μg/mL) and peak areas (*y*) of each compound were expressed by the regression equations (*y* = a*x* + b) (Table 2 and Supplementary Figure 1). Calibration curves of the marker compounds revealed good linearity (*r*^2^ ≥ 0.9998). The LODs and LOQs for the tested compounds were 0.015–0.925 and 0.046–2.804 μg/mL, respectively.

### 2.3. Determination of the Seven Marker Compounds of the UGS Extract

The established analytical method using HPLC was used to the simultaneous quantification of the seven marker compounds of UGS extract. The amounts of the seven marker compounds ranged from 0.190 mg/g to 16.431 mg/g. As shown in Table 3, decursin (16.431 mg/g) was the most abundant compound among these seven compounds.

### 2.4. Precision, Accuracy, and Recovery

Precision was represented as the relative standard deviations (RSDs) of the concentrations of marker compounds in mixed standard solutions, and repeated five times at low, middle and high concentration levels. The results for the intra- and inter-day precision and accuracy are shown in Table 4 The intra- and inter-day precision for the seven marker compounds in mixed standard solutions was <3.36%, and the accuracy ranged from 95.12% to 105.12%. The RSD values for repeatability for the seven compounds ranged from 0.16% to 0.55% for retention times and from 0.4% to 0.76% for peak areas (Table 5). Recovery tests of the seven marker compounds were performed by adding the three different known amounts (low, middle, and high concentrations) of standard solutions to a certain amount of UGS extract. The recoveries of the seven marker compounds were between 95.99% and 104.94%, with RSD ≤ 3.21% (Table 6). These results demonstrate that the established method of HPLC had satisfactory precision, accuracy, repeatability, and recovery for simultaneous analysis.

### 2.5. Antioxidant Activity of the Marker Compounds of UGS

**Figure 3:**
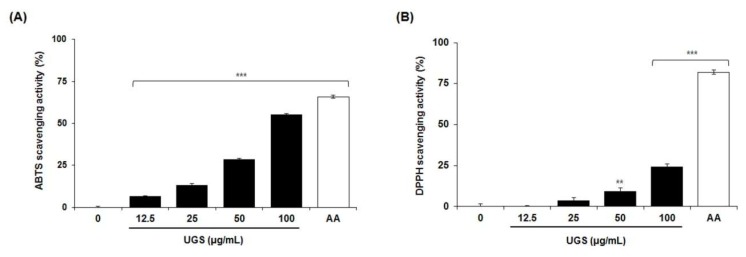
Effects of UGS on free radical-scavenging activities. The antioxidant activity of UGS against ABTS (A) or DPPH (B) was assessed using a radical-scavenging method. The quantitative data are presented as the mean ±SEM of triplicate experiments. ** *P* < 0.01” or *** *P* < 0.001 vs vehicle control cells *n* = 3/sample. ‘0′ in x-axis represents vehicle control. AA: L-ascorbic acid, a positive control.

**Figure 4:**
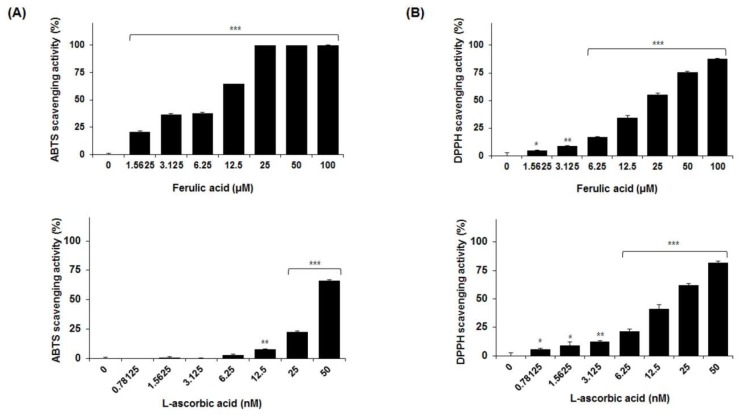
Effects of ferulic acid on free radical-scavenging activities. The antioxidant activity of different concentrations of ferulic acid and L-ascorbic acid against ABTS (A) or DPPH (B), as assessed using a radical-scavenging method. _L_-ascorbic acid was used as a positive control of antioxidant. The quantitative data are presented as the mean ± SEM of triplicate experiments. * *P* < 0.05, ** *P* < 0.01 or *** *P* < 0.001 vs vehicle control cells n = 3/sample.

To assess the antioxidant activity of UGS, the current study measured their scavenging activities against the 2,2′-azinobis-(3-ethylbenzothiazoline-6-sulfonic acid) (ABTS) and 2,2′-diphenyl-1-picrylhydrazyl (DPPH) radicals. As shown in Figure 3A,B, UGS extract dose-dependently increased the ABTS and DPPH radical scavenging activities. The effect on DPPH was not significant compared to ABTS. Then, antioxidant activity of the seven marker compounds of UGS was tested. These results revealed that ferulic acid dramatically increased the scavenging activity for ABTS in a dose-dependent manner. The concentration of ferulic acid required for a 50% reduction (RC_50_) in ABTS radicals was 16.22 μM (Table 7 and Figure 4A). The antioxidant activities obtained for ferulic acid using the DPPH assay are shown in Table 8 and Figure 4B. Similar to the results observed for ABTS, ferulic acid reduced the formation of the DPPH radical in a dose-dependent manner. The RC_50_ of ferulic acid against DPPH radicals was 41.21 μM. L-ascorbic acid was used as a positive antioxidant control compound.

### 2.6. Biological Activitis of the UGS Extract in Neuronal Cell Lines

**Figure 5:**
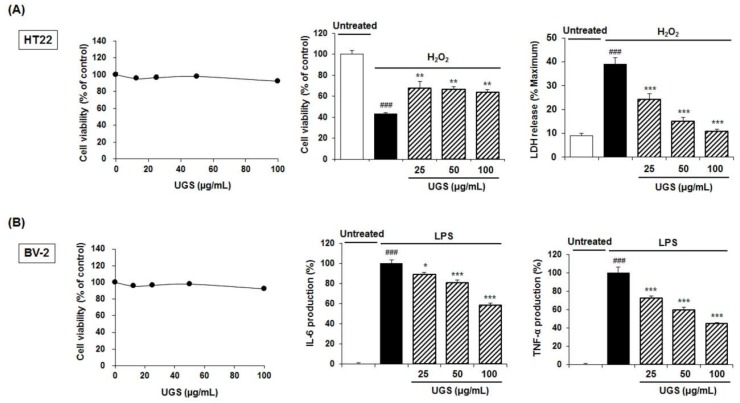
Biological effects of UGS extract on neuroprotection and anti-inflammation in HT22 neuronal hippocampal cells and BV-2 microglia. (A) Cell viability was performed to assess the cytotoxicity of HT22 cells against UGS extract using the cell counting Kit (CCK)-8 assay. Neuroprotective activity of UGS was tested using the CCK assay (middle) and LDH release assay (right). The results are expressed as the mean ±SEM of three independent experiments. ^###^ *P* < 0.001 vs vehicle control cells; *** *P* < 0.001 and ** *P* < 0.01 vs H_2_O_2_-treated cells. (B) Cell viability was performed to assess the cytotoxicity of BV-2 cells against UGS extract using the CCK-8 assay. The UGS effect on lipopolysaccharide (LPS)-induced production of proinflammatory cytokines were assessed in BV-2 cells using ELISA. Cells were pretreated with UGS for 2 h and then stimulated with LPS for an additional 22 h. The results are expressed as the mean ± SEM of three independent experiments. ^###^ *P* < 0.001 vs vehicle control cells; *** *P* < 0.001, ** *P* < 0.01 and * *P* < 0.05 vs LPS-treated cells.

The current study examined the biological effects of the UGS extract on the neurodegenerative diseases. The cell viability test was carried out to measure the viability of HT22 hippocampal cells against UGS extract. Treatment with UGS extract did not affect the cell viability at ≤100 μg/mL (Figure 5A). Then, the neuroprotection activity of UGS was investigated. HT22 cells were stimulated with H_2_O_2_ in the absence or presence of the UGS extract. H_2_O_2_ treatment significantly decreased the viability of HT22 whereas UGS extract significantly reversed the H_2_O_2_-mediated cell death. The generation of lactose dehydrogenase (LDH) was further confirmed the neuroprotective effect of UGS. Consistent with the results of viability assay, H_2_O_2_ stimulation significantly enhanced the release of LDH compared with the untreated cells. In contrast, the UGS extract significantly blocked the release of LDH in H_2_O_2_-stimulated HT22 cells compared with the cells treated with H_2_O_2_ alone (Figure 5A).

Additionally, the inhibitory effect of UGS on neuroinflammation was studied using microglia cell line. The cell viability assay was performed to assess cytotoxicity of UGS against BV-2 cells. As shown in Figure 5B, no cytotoxicity of UGS extract was observed up to 100 μg/mL. Subsequent experiments were conducted at the range of nontoxic concentrations. To investigate the effects of UGS on the pro-inflammatory cytokine production, ELISAs for tumor necrosis factor-alpha (TNF-α) and interleukin-6 (IL-6) was performed using culture supernatant from the lipopolysaccharide (LPS)-stimulated BV-2 cells. Results showed that stimulation with LPS- significantly increased TNF-α and IL-6 levels. In contrast, UGS treatment significantly reversed the LPS effect on production of TNF-α and IL-6 (Figure 5B).

## 3. Discussion

In these study, a simultaneous analysis of the seven marker compounds of UGS was performed using the HPLC-PDA method. The main ingredients of each medicinal herb forming UGS, are as follows: alkaloids (e.g., corynoxeine and hirsutine) from *Uncaria sinensis* [21], sesquiterpenes (e.g., atractylon and atractylenolide I-III) from *Atractylodes japonica* [22], triterpenes (e.g., pachymic acid, dehydrotumulosic acid, and dehydrotrametenolic acid) from *Poria cocos* [23], triterpene saponins (e.g., saikosaponin A, C, and D) from *Bupleurum falcatum* [24], coumarins (e.g., decursin, decursinol angelate, and nodakenin) from *Angelica gigas* [25], alkylphthalides (e.g., cnidilide, ligustilide, butylphthalide, and neocnidilide) and phenol (e.g., ferulic acid) from *Cnidium officinale* [26,27], and flavonoids (e.g., liquiritin, liquiritin apioside and liquiritigenin) and triterpene saponins (e.g., glycyrrhizin) from *Glycyrrhiza uralensis* [28]. Among the various ingredients, the current study conducted simultaneous determination of the seven components liquiritin apioside, liquiritin, glycyrrhizin (*Glycyrrhiza uralensis*), ferulic acid (*Cnidium officinale*), decursin, decursinol angelate (*Angelica gigas*), and atractylenolide I (*Atractylodes japonica*) in the formulation of UGS by the established and validated analytical HPLC-PDA method. Consequently, decursin (16.431 mg/g), marker compound of *Angelica gigas*, was found as major compound in the UGS.

There are increasing evidence on the powerful antioxidant activity of herbal medicines [29,30]. Herbal medicines contain various free radical scavenging molecules that mediate oxidative stress, which ultimately causes a variety of diseases such as cancer, inflammation, and metabolic disorders [31,32,33]. Thus, antioxidant therapy is considered as an attractive approach to treat various human diseases [34,35]. To date, previous studies reported significant antioxidant activities of various herbal formulas such Galkeun-tang [36] and Samchulgeonbi-tang [37] by regulating the free radical scavenging activity were reported. The current study data revealed that UGS extract activate the free radical scavenging effects against ABTS and DPPH. Among seven marker compounds, ferulic acid showed antioxidant activity compared with others. The scavenging activities of UGS extract were 100 ± 0.1% and 87.8 ± 0.5% for ABTS and DPPH, respectively, at 100 μg/mL. In contrast, the activities of other six compounds were below 43.6% and 4.1% for ABTS and DPPH, respectively, at 100 μg/mL. These results suggest that ferulic acid may be an active compound of UGS possessing the potent antioxidant activity.

Similarly, Liang et al. reported in vitro antioxidant effect of ferulic acid from *Spiranthes sinensis* [38]. Additionally, to investigate the biological activity of UGS on the neurodegenerative diseases, neuronal cell lines were treated with various concentrations of UGS extract in the presence of neuronal damage inducers H_2_O_2_ and LPS, respectively. The results demonstrated that UGS has the effects on neuroprotection and anti-neuroinflammation in vitro. Further studies are required to verify the UGS effect using additional in vitro and in vivo neuronal diseases-related models. 

In conclusion, the current study established a HPLC method for the quantitative analysis of the seven marker compounds present in extracts of UGS. Validation results of the method displayed good linearity, repeatability, intra- and inter-day precision, and recovery, indicating a successful application for the simultaneous analysis of marker compounds for the quality control of UGS. In addition, the results of antioxidant activity assays demonstrate the potent antioxidant activity of ferulic acid as an active compound of UGS.

## 4. Materials and Methods

### 4.1. Plant Materials

The seven crude herbal medicines forming UGS, Uncariae Ramulus et Uncus, Atractylodis Rhizoma Alba, Poria Sclerotium, Bupleuri Radix, Angelicae Gigantis Radix, Cnidii Rhizoma, and Glycyrrhizae Radix et Rhizoma, were purchased at the Kwangmyungdang herbal market (Ulsan, South Korea). Voucher specimens (SCD-B-032) have been deposited at the Clinical Medicine Division, Korea Institute of Oriental Medicine.

### 4.2. Chemicals and Reagents

The marker compound glycyrrhizin was purchased from ChemFaces Biochemical Co., Ltd. (Wuhan, China); atractylenolide I, ferulic acid, and decursinol angelate were purchased from Biopurify Phytochemicals (Chengdu, China); and liquiritin apioside, liquiritin, and decursin were purchased from Sunny Biotech Co., Ltd. (Shanghai, China). The chemical structures of compounds are shown in Figure 1. The purity of them was ≥98.0% according to HPLC analysis. HPLC-grade acetonitrile, methanol, and water were purchased from J. T. Baker Chemical Co. (Phillipsburg, NJ, USA), and analytical-grade acetic acid was purchased from Sigma-Aldrich (St. Louis, MO, USA).

### 4.3. Apparatus and Chromatographic Conditions

A Waters Alliance e2695 HPLC system (Waters Corp., Milford, MA, USA) equipped with a pump, degasser, column oven, auto sample injector, and photodiode array (PDA) detector (#2998, Waters Corp. Milford, MA, USA) was used in the quantitative analysis and Empower software (version 3; Waters Corp, Milford, MA, USA) was used to data processing. The chromatographic separation of the seven marker compounds was performed at 30 °C using a Gemini C-18 analytical column (250 × 4.6 mm, 5 μm; Phenomenex, Torrance, CA, USA) with a gradient solvent system of 1.0% (*v*/*v*) aqueous acetic acid (A) and acetonitrile (B). The elution conditions were as follows: 12–42% B for 0–25 min, 42–52% B for 25–30 min, 52–65% B for 30–55 min, 65–100% B for 55–56 min, and 100% B for 56–63 min. The flow rate and injection volume were 1.0 mL/min and 10 μL, respectively. The wavelength range of the PDA detector was 190 nm to 400 nm.

### 4.4. Preparation of Standard Solutions

The seven marker compounds were dissolved in methanol at a concentration of 1.0 mg/mL. Then, these stock solutions were diluted to make series of standard solutions with different concentrations for quantitative analysis.

### 4.5. Preparation of the UGS 70% Ethanol Extract and Sample Solutions

The UGS composed of the seven crude herbal medicines, Uncariae Ramulus et Uncus, Atractylodis Rhizoma Alba, Poria Sclerotium, Bupleuri Radix, Angelicae Gigantis Radix, Cnidii Rhizoma, and Glycyrrhizae Radix et Rhizoma, was mixed as indicated in Table 8 (41 g) and extracted using 70% aqueous ethanol (twice each with 246 mL) by refluxing for 2 h at 100 °C. The 70% ethanol extract was then filtered through a filter paper (5 μm) and concentrated using a rotary evaporator system (EYELA N-1000, Rikakikai Co., Tokyo, Japan) under vacuum to make powdered extract (8.613 g). The yield of UGS extract was 21%. The extract of UGS was weighed accurately and dissolved in methanol at 10 mg/ml for simultaneous determination. Then, the sample solution was filtered through a syringe filter (0.45 μm) and used for HPLC analysis. For testing biological activities, the extract of UGS was dissolved in dimethylsulfoxide (DMSO).

### 4.6. Calibration Curve, LOD, and LOQ

The calibration curves of compounds were calculated from the peak areas of the standard solutions at different concentrations. The concentration ranges of marker compounds were as follows: liquiritin apioside (3.125–50 μg/mL), liquiritin (1.5625–25 μg/mL), ferulic acid (0.78125–25 μg/mL), glycyrrhizin and decursinol angelate (6.25–200 μg/mL), decursin (12.5–400 μg/mL), and atractylenolide I (0.78125–12.5 μg/mL). These solutions were measured in triplicate for the preparation of the calibration curves. The slope of the calibration curve and the standard deviation (SD) of the intercept were used to calculate the LOD and LOQ for the seven marker compounds, as follows:

LOD = 3.3 × (SD of the response/slope of the calibration curve)(1)

LOQ = 10 × (SD of the response/slope of the calibration curve)(2)

### 4.7. Precision, Accuracy, and Recovery

To evaluate the precision of the established HPLC conditions, intra- and inter-day variations were measured using the mixed standard solutions with low, middle, and high concentration levels of marker compounds. For the measurement of intra-day precision and accuracy, the mixed standard solutions were analyzed five times in a single day. The inter-day precision was assessed by repeating the analysis of the mixed standard solutions for three consecutive days. The intra- and inter-day RSD (%) was used to express the precision, and the percentage of the observed concentration for the fortified concentration were used to present the accuracy. To confirm the repeatability, six replicates were measured using the mixed standard solutions and the RSDs of retention times and peak areas for each compound were used. The recoveries of the seven compounds were determined by adding standard solutions at three different concentration levels (low, middle, and high) to the extract of UGS samples (100 mg for liquiritin apioside, liquiritin, ferulic acid, and atractylenolide I, and 10 mg for glycyrrhizin, decursin, and decursinol angelate); the 90% aqueous methanol was added to volume metric flask to make 10mL sample solution. The recovery was performed five times at each level and calculated as follows:

(3) $$\mathrm{Recovery}\;\left(\%\right)=\frac{\mathrm{found}\;\mathrm{concentration}-\;\mathrm{original}\;\mathrm{concentration}}{\mathrm{spiked}\;\mathrm{concentration}}\;\times \;100$$

### 4.8. ABTS-Scavenging Activity

The ABTS radical-scavenging activity of the UGS extract was assessed according to the previous study [36]. The ABTS radical cation was prepared by reaction with a 7 mM ABTS solution and 2.45 mM potassium persulfate, followed by keeping in the dark at room temperature for 16 h. The absorbance of the reaction mixture was adjusted to 0.7 at 734 nm. A 100 μL of sample solution at various concentrations (12.5–200 µg/mL) was mixed with ABTS^•+^ solution. The mixture was incubated in the dark at room temperature for 5 min and the absorbance at 734 nm was measured using a spectrophotometer (Benchmark Plus, Bio-Rad, CA, USA). The ABTS radical-scavenging capacity of the UGS extract was measured using the following equation:

(4) $$\mathrm{ABTS}\;\mathrm{scavenging}\;\mathrm{activity}\;\left(\%\right)=\frac{1-\mathrm{absorbance}\;\mathrm{at}\;734\;\mathrm{nm}\;\mathrm{of}\;\mathrm{UGS}\;\mathrm{extract}}{\mathrm{absorbance}\;\mathrm{at}\;734\;\mathrm{nm}\;\mathrm{of}\;\mathrm{control}}\;\times \;100$$

### 4.9. DPPH-Scavenging Activity

The DPPH radical-scavenging activity of the UGS extract was assessed according to the previous study [37]. In brief, a 100 μL aliquot of sample solution at various concentrations was mixed with 100 μL of 0.15 mM DPPH solution in methanol. The mixture was incubated in the dark at room temperature for 30 min and the absorbance at 517 nm was measured on a spectrophotometer (Benchmark Plus, Bio-Rad, CA, USA). The DPPH radical-scavenging capacity of the UGS extract was measured using the following equation:

(5) $$\mathrm{DPPH}\;\mathrm{scavenging}\;\mathrm{activity}\;\left(\%\right)=\frac{1-\mathrm{absorbance}\;\mathrm{at}\;517\;\mathrm{nm}\;\mathrm{of}\;\mathrm{UGS}\;\mathrm{extract}}{\mathrm{absorbance}\;\mathrm{at}\;517\;\mathrm{nm}\;\mathrm{of}\;\mathrm{control}}\;\times \;100$$

### 4.10. Cell Lines and Culture

HT22 and BV-2 cells were maintained in Dulbecco’s modified Eagle’s medium (Hyclone/Thermo, Rockford, IL, USA) supplemented with 10% fetal bovine serum (Hyclone/Thermo, Rockford, IL, USA) and penicillin/streptomycin in 5% CO_2_ at 37 °C.

### 4.11. Cell Counting Kit (CCK) Assay

Cells were plated on 96-well microplates and treated with various concentrations of UGS extract in DMSO for 24 h. After adding CCK-8 solution (Dojindo, Kumamoto, Japan) to each well, and the cells were maintained for 4 h at 37 °C. The absorbance at 450 nm was measured on an Epoch microplate spectrophotometer (Bio-Tek Instruments, Inc., Winooski, VT, USA). The cell viability was determined using the following equation:

(6) $$\mathrm{Cell}\;\mathrm{viability}\;\left(\%\right)=\frac{\mathrm{Mean}\;\mathrm{OD}\;\mathrm{in}\;\mathrm{UGS}\;\mathrm{extract}-\mathrm{treated}\;\mathrm{cells}}{\mathrm{Mean}\;\mathrm{OD}\;\mathrm{in}\;\mathrm{untreated}\;\mathrm{cells}}\;\times \;100$$

To examine the neuroprotective effect of UGS extract, HT22 cells were co-treated with UGS and H_2_O_2_ (Sigma-Aldrich, St. Louis, MO, USA) for 6 h.

### 4.12. LDH Release Assay

The release of LDH was measured using the CytoTox 96 nonradioactive cytotoxicity assay kit (Promega, Madison, WI, USA). Cell lysates and supernatants were prepared to induce maximal LDH release and experimental LDH release, respectively, and incubated with substrate mixture in the dark at room temperature for 30 min. Stop solution was added to each well and absorbance at 490 nm was determined on an Epoch microplate spectrophotometer (Bio-Tek Instruments, Inc., Winooski, VT, USA). The cytotoxicity of the UGS extract was calculated using the following formula:

(7) $$\mathrm{Cytotoxicity}\;\left(\%\right)=\frac{\mathrm{Experimental}\;\mathrm{LDH}\;\mathrm{release}\;}{\mathrm{Maximum}\;\mathrm{LDH}\;\mathrm{release}}\;\times \;100$$

### 4.13. Enzyme-Linked Immunosorbent Assays (ELISAs) for Cytokine Production

BV-2 cells were pretreated with UGS extract for 2 h and treated with LPS (1 μg/mL) for an additional 22 h. Culture supernatants were collected and the levels of TNF-α and IL-6 were assessed using ELISA kits from R&D Systems (Minneapolis, MN, USA).

### 4.14. Statistical Analysis

The data are expressed as the mean ±SEM. Data were analyzed using one-way analysis of variance and Dunnett’s multiple comparisons test and student’s t-test. *P* < 0.05 was considered significant.

## Figures and Tables

**Table 1:** Composition of UGS.

Latin Name	Scientific Name	Amount (g)	Origin
Uncariae Ramulus et Uncus	*Uncaria sinensis*	6	China
Atractylodis Rhizoma Alba	*Atractylodes japonica*	8	China
Poria Sclerotium	*Poria cocos*	8	China
Bupleuri Radix	*Bupleurum falcatum*	4	China
Angelicae Gigantis Radix	*Angelica gigas*	6	Bonghwa, Korea
Cnidii Rhizoma	*Cnidium officinale*	6	China
Glycyrrhizae Radix et Rhizoma	*Glycyrrhiza uralensis*	3	China
Total amount		41	

**Table 2:** Linear range, regression equation, correlation coefficients, LODs, and LOQs for compounds.

Compound.	Linear Range (μg/mL)	Regression Equation (*y* = a*x* + b) ^a)^		*r* ^2^	LOD ^b)^ (μg/mL)	LOQ ^c)^ (μg/mL)
		Slope (a)	Intercept (b)			
Liquiritin apioside	3.125–50	15290	4536.9	1.0000	0.177	0.537
Liquiritin	1.5625–25	18759	2614.9	0.9999	0.052	0.157
Ferulic acid	0.78125–25	56995	1865.6	1.0000	0.039	0.118
Glycyrrhizin	6.25–200	4882.5	1533	1.0000	0.619	1.876
Decursin	12.5–400	30409	77457	0.9998	0.925	2.804
Decursinol angelate	6.25–200	35125	38848	0.9998	0.232	0.705
Atractylenolide I	0.78125–12.5	62615	1322.9	1.0000	0.015	0.046

**^a^**^)^*y* = a*x* + b, y means peak area and *x* means concentration (μg /mL). **^b)^** LOD (Limit of detection): 3.3 × (SD of the response/slope of the calibration curve). **^c)^** LOQ (Limit of quantitation): 10 × (SD of the response/slope of the calibration curve).

**Table 3:** The content of marker compounds in UGS.

Compound	Content (mg/g)
Liquiritin apioside	1.671 ± 0.004
Liquiritin	2.014 ± 0.004
Ferulic acid	0.605 ± 0.002
Glycyrrhizin	10.267 ± 0.05
Decursin	16.431 ± 0.04
Decursinol angelate	7.606 ± 0.002
Atractylenolide I	0.190 ± 0.001

**Table 4:** Precision and accuracy of seven marker compounds in UGS.

Compound	Fortified Conc. (μg/mL)	Intra-Day (n = 5)			Inter-Day (n = 5)		
		Observed Conc. (μg/mL)	Precision ^a)^ (%)	Accuracy ^b)^ (%)	Observed Conc. (μg/mL)	Precision (%)	Accuracy (%)
Liquiritin apioside	5	4.84	2.11	96.88	5.01	1.25	100.18
	10	10.14	1.49	101.36	10.19	0.96	101.87
	20	20.10	0.96	100.50	20.15	0.61	100.76
Liquiritin	5	4.99	2.52	99.78	5.09	0.74	101.84
	10	10.50	1.65	105.04	10.36	1.14	103.63
	20	20.49	1.13	102.46	20.44	0.73	102.18
Ferulic acid	1.5	1.46	3.36	97.63	1.47	2.73	97.81
	3	3.15	1.36	105.12	2.97	2.98	98.93
	6	6.28	0.84	104.63	5.85	2.90	97.42
Glycyrrhizin	12.5	12.26	0.97	98.08	12.25	0.96	97.98
	25	24.98	2.00	99.92	24.47	0.86	97.90
	50	49.44	0.38	98.88	49.51	0.42	99.02
Decursin	20	19.74	1.42	98.71	19.68	1.48	98.42
	40	41.51	0.97	103.78	40.47	1.22	101.17
	80	81.37	0.72	101.71	81.34	0.53	101.67
Decursinol angelate	10	9.58	0.93	95.76	9.51	2.61	95.12
	20	19.55	0.73	97.73	19.50	0.45	97.51
	40	39.13	0.43	97.83	39.11	0.46	97.78
Atractylenolide I	1	0.98	2.53	98.40	1.00	1.14	100.07
	2	2.05	1.48	102.60	2.02	0.95	101.23
	4	4.02	0.98	100.50	4.01	0.69	100.37

**^a^**^)^ Precision is expressed as RSD (%) = (SD/Mean) × 100. ^b)^ Accuracy (%) = (Observed concentration/Fortified concentration) × 100.

**Table 5:** Repeatability of retention times and peak areas for the seven analytes (n = 6).

Compound	Retention Time (min)		Peak Area (AU)	
	Mean ± SD	RSD (%)	Mean ± SD	RSD (%)
Liquiritin apioside	12.48 ± 0.05	0.43	234009.83 ± 1419.74	0.61
Liquiritin	13.00 ± 0.06	0.49	143858.00 ± 903.60	0.63
Ferulic acid	14.09 ± 0.08	0.55	212067.33 ± 1604.31	0.76
Glycyrrhizin	33.46 ± 0.18	0.52	144019.50 ± 726.13	0.50
Decursin	42.93 ± 0.07	0.16	1905525.83 ± 12044.19	0.63
Decursinol angelate	43.45 ± 0.07	0.16	1094712.83 ± 4431.44	0.40
Atractylenolide I	50.27 ± 0.12	0.24	234499.67 ± 1056.72	0.45

SD: Standard deviation; RSD: Relative standard deviation.

**Table 6:** Recovery of seven marker compounds in UGS.

Compound	Original Conc. (μg/mL)	Spiked Conc. (μg/mL)	Found Conc. (μg/mL)	Recovery ^a)^ ± SD (%)	RSD (%)
Liquiritin apioside	17.75	4	21.59	95.99 ± 0.75	0.78
		10	27.86	101.14 ± 1.34	1.33
		20	37.56	99.07 ± 1.28	1.29
Liquiritin	21.27	4	25.46	104.94 ± 0.84	0.80
		10	31.61	103.49 ± 1.72	1.66
		20	41.42	100.80 ± 1.41	1.40
Ferulic acid	6.63	1.5	8.16	101.49 ± 1.55	1.53
		3	9.73	103.14 ± 0.76	0.74
		6	12.79	102.70 ± 0.61	0.60
Glycyrrhizin	9.21	2.5	11.63	97.04 ± 1.32	1.36
		5	14.08	97.45 ± 1.21	1.24
		10	18.90	96.94 ± 1.40	1.44
Decursin	14.81	4	18.84	100.68 ± 3.23	3.21
		8	22.56	96.77 ± 1.05	1.08
		16	30.48	97.91 ± 0.75	0.77
Decursinol angelate	6.90	2	8.85	97.26 ± 1.16	1.20
		4	10.76	96.53 ± 0.47	0.48
		8	15.22	104.00 ± 0.35	0.33
Atractylenolide I	2.08	1	3.11	102.93 ± 1.30	1.26
		2	4.14	102.68 ± 1.49	1.46
		4	5.97	97.24 ± 0.42	0.43

**^a^**^)^ Recovery (%) = (Found concentration – Original concentration)/spiked concentration × 100.

**Table 7:** ABTS/DPPH radical scavenging activity of marker compounds of UGS.

μM	1	2	3	4	5	6	7	nM	L-ascorbicAcid *
ABTS									
0	0.0 ± 0.9	0.0 ± 0.9	0.0 ± 0.9	0.0 ± 0.9	0.0 ± 0.9	0.0 ± 0.9	0.0 ± 0.9	0	0.0 ± 0.9
1.5625	2.3 ± 0.6	1.9 ± 0.3	20.8 ± 0.7	0.7 ± 0.9	0.1 ± 0.3	–0.2 ± 0.3	–1.1 ± 0.6	0.78125	–0.9 ± 0.9
3.125	3.2 ± 0.6	3.5 ± 0.5	36.7 ± 0.4	0.0 ± 0.8	0.3 ± 0.2	–1.1 ± 0.2	2.2 ± 1.3	1.5625	0.8 ± 0.6
6.25	7.0 ± 0.6	5.5 ± 0.4	38.0 ± 0.8	–0.1 ± 0.7	0.7 ± 0.6	0.0 ± 0.2	–0.4 ± 1.1	3.125	-0.2 ± 0.5
12.5	10.1 ± 0.3	10.5 ± 0.1	64.4 ± 0.2	–0.8 ± 0.9	–1.5 ± 0.7	–0.3 ± 0.2	0.4 ± 0.6	6.25	2.7 ± 0.8
25	18.8 ± 0.8	19.7 ± 0.8	99.7 ± 0.1	1.3 ± 0.1	0.1 ± 0.4	–0.4 ± 0.6	1.0 ± 1.1	12.5	7.4 ± 0.5
50	28.3 ± 0.4	29.0 ± 0.6	99.8 ± 0.0	2.7 ± 1.0	0.5 ± 0.9	1.5 ± 0.2	2.0 ± 0.5	25	22.4 ± 0.6
100	43.6 ± 0.0	43.1 ± 0.7	100.0 ± 0.1	3.6 ± 0.2	0.0 ± 0.1	2.6 ± 0.3	–0.7 ± 0.3	50	65.9 ± 1.0
DPPH									
0	0.0 ± 2.5	0.0 ± 2.5	0.0 ± 2.5	0.0 ± 2.5	0.0 ± 2.5	0.0 ± 2.5	0.0 ± 2.5	0	0.0 ± 2.5
1.5625	0.9 ± 0.2	0.9 ± 0.2	4.8 ± 0.5	–0.1 ± 0.7	0.9 ± 0.7	–0.2 ± 1.0	2.7 ± 0.4	0.78125	5.7 ± 1.1
3.125	–0.2 ± 0.3	–0.2 ± 0.3	8.6 ± 0.5	–0.7 ± 0.7	0.1 ± 1.4	3.7 ± 1.0	–0.5 ± 0.5	1.5625	9.1 ± 3.0
6.25	2.5 ± 1.0	2.5 ± 1.0	16.9 ± 0.1	–1.7 ± 1.2	0.3 ± 1.3	2.3 ± 0.5	0.4 ± 0.8	3.125	12.4 ± 1.0
12.5	2.8 ± 0.5	2.8 ± 0.5	34.6 ± 2.0	–0.9 ± 0.6	0.6 ± 0.8	3.6 ± 1.4	1.0 ± 0.7	6.25	21.3 ± 1.0
25	1.7 ± 0.7	1.7 ± 0.7	55.1 ± 1.7	0.5 ± 1.3	0.5 ± 0.3	5.5 ± 1.4	1.3 ± 1.0	12.5	41.5 ± 3.6
50	4.1 ± 1.2	4.1 ± 1.2	75.5 ± 0.9	1.9 ± 0.9	0.4 ± 0.2	4.1 ± 1.2	3.1 ± 0.5	25	61.8 ± 1.4
100	2.2 ± 1.3	2.2 ± 1.3	87.8 ± 0.5	4.1 ± 0.4	0.2 ± 1.6	0.9 ± 1.6	–1.8 ± 0.6	50	82.0 ± 1.2

Liquiritin apioside (1), liquiritin (2), ferulic acid (3), glycyrrhizin (4), decursin (5), decursinol angelate (6), and atractylenolide I (7); * L-ascorbic acid was used as a positive control of antioxidant; The quantitative data are presented as the mean ± SEM of triplicate experiments.

**Table 8:** Composition of UGS.

Latin Name	Scientific Name	Amount (g)	Origin
Uncariae Ramulus et Uncus	*Uncaria sinensis*	6	China
Atractylodis Rhizoma Alba	*Atractylodes japonica*	8	China
Poria Sclerotium	*Poria cocos*	8	China
Bupleuri Radix	*Bupleurum falcatum*	4	China
Angelicae Gigantis Radix	*Angelica gigas*	6	Bonghwa, Korea
Cnidii Rhizoma	*Cnidium officinale*	6	China
Glycyrrhizae Radix et Rhizoma	*Glycyrrhiza uralensis*	3	China
Total amount		41	

## Supplementary Materials

Supplementary Figure 1 is available online.
