# Supplementary material for: Simultaneous Determination of the Traditional Herbal Formula Ukgansan and the In Vitro Antioxidant Activity of Ferulic Acid as an Active Compound
Source: Molecules. 2018 Jul 7;23(7):1659. doi: 10.3390/molecules23071659 (PMC6100485; doi:10.3390/molecules23071659)

Supplementary figure 1. Calibration curves, LODs and LOQs of seven marker compounds in UGS. The regression equations are presented as the mean of triplicate experiments. LODs and LOQs are calculated by 3.3 × (SD of the response/slope of the calibration curve) and 10 × (SD of the response/slope of the calibration curve), respectively.

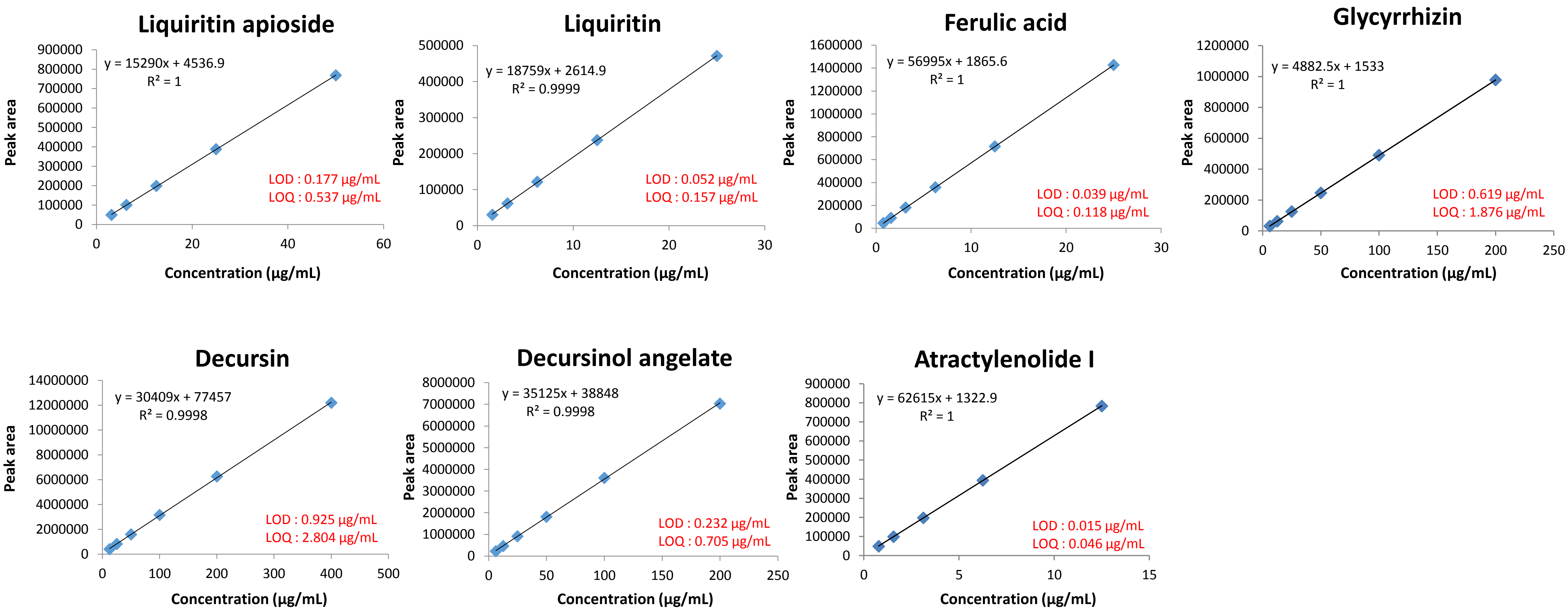

Supplement: Supplementary file 1 [file molecules-23-01659-s001.pdf]
